# Endometriosis Is Associated with Increased Serum and Peritoneal Fluid Concentrations of Chromogranin A and Its Derivatives

**DOI:** 10.3390/jcm15041567

**Published:** 2026-02-16

**Authors:** Alicja Sztokfisz-Ignasiak, Maja Owe-Larsson, Maciej Maj, Hubert Rytel, Kateryna Shevchenko, Filip Dąbrowski, Piotr Laudański, Mikołaj Pater, Izabela Róża Janiuk, Jacek Malejczyk

**Affiliations:** 1 Department of Histology and Embryology, Centre of Biostructure Research, Medical University of Warsaw, 02-004 Warsaw, Poland; alicja.sztokfisz-ignasiak@wum.edu.pl (A.S.-I.); maja.owe-larsson@wum.edu.pl (M.O.-L.);; 2 Hospital Pharmacy, National Medical Institute of the Ministry of Interior and Administration, 02-507 Warsaw, Poland; 3 Department of Gynaecology and Gynaecological Oncology, Medical Centre of Postgraduate Medical Education CMKP, 01-813 Warsaw, Poland; 4 Department of Obstetrics, Gynaecology and Gynaecological Oncology, Medical University of Warsaw, 03-242 Warsaw, Poland; 5 Women’s Health Research Institute, Calisia University, 62-800 Kalisz, Poland; 6 OVIklinika Infertility Center, 01-377 Warsaw, Poland; 7 Institute of Health Sciences, Faculty of Medical and Health Sciences, University of Siedlce, 08-110 Siedlce, Poland

**Keywords:** endometriosis, non-invasive markers, chromogranin A, catestatin, pancreastatin, inflammation, serum, peritoneal fluid

## Abstract

**Background/Objectives**: Endometriosis is a prevalent gynecological illness associated with chronic pain, inflammation, and infertility, as ectopic endometrial lesions are formed. No fully effective treatment is available, and the pathogenesis of this disease is unclear. The survival of ectopic endometrial cells is facilitated by their low susceptibility to apoptosis, an immunosuppressive environment, and local angiogenesis. Chromogranin A (CgA), a glycoprotein prohormone, modulates various processes including angiogenesis and innate immunity, and its higher levels are detected in neuroendocrine tumors and inflammatory disorders. Since endometriosis may be considered an autoinflammatory disorder, this study aimed to evaluate serum and peritoneal fluid concentrations of CgA and its derivatives, catestatin and pancreastatin, and to correlate these levels with disease severity. **Methods**: The study was conducted on samples of serum and peritoneal fluid (PF) obtained from 65 women diagnosed with endometriosis and from 60 control individuals who underwent surgery for other reasons. The concentrations of CgA, catestatin, and pancreastatin were assessed in the collected samples by specific enzyme-linked immunosorbent assays. **Results**: CgA, catestatin, and pancreastatin concentrations were significantly higher in the sera and PF of endometriosis patients compared to controls. There was a correlation between their serum and PF levels, and all tested factors were correlated with each other in both serum and PF. Serum concentrations of CgA, catestatin, and pancreastatin were also associated with disease progression. Receiver operating characteristic (ROC) analysis further confirmed that endometriosis is associated with increased circulating CgA, catestatin, and pancreastatin levels, suggesting that they may be considered markers of endometriosis. **Conclusions**: The upregulation of CgA and its derivatives in endometriosis may indicate their role in the disease pathogenesis and implicate them as potential diagnostic markers and/or therapeutic targets.

## 1. Introduction

Endometriosis, a chronic disease that affects approximately 10% of women of reproductive age, is an estrogen-dependent gynecological disorder related to the persistence of endometrial-like tissue (glands and stroma) outside the uterus [1]. Endometriotic lesions predominantly localize in the peritoneal cavity and may present as ovarian endometriotic cysts, superficial peritoneal lesions, or deep infiltrating lesions, including those of the rectovaginal septum [2,3,4,5]. The persistence of active lesions is associated with chronic pelvic inflammatory disease, which may account for local fibrosis, adhesion formation, and pain [6,7]. The primary clinical symptoms include dysmenorrhea, chronic pelvic pain, and dyspareunia. It is estimated that up to 50% cases of female infertility are linked with endometriosis [8]. Pelvic pain may affect patients’ working capabilities and mental health, being responsible for anxiety and depression. Thus, endometriosis is a debilitating condition that may seriously affect the quality of life [9,10].

So far, there is no fully effective treatment for endometriosis. Therefore, the disease constitutes a significant clinical, social, and socio-economic problem.

The origin and etiopathology of endometriosis remain poorly understood. It is assumed that endometriotic cells may be relocated to the peritoneal cavity in the course of retrograde menstruation [11] and, occasionally, by the lymphatic or blood circulatory system, which may explain a distant localization of some lesions, e.g., in the thorax or brain [12]. It cannot be excluded that some forms of endometriosis may also originate from coelomic metaplasia or Müllerian system remnants [13].

The reason for the survival and growth of ectopic endometrial cells remains an unanswered question. It is plausible that this may be due to a permissive, immunosuppressive local peritoneal milieu and impaired elimination of endometriotic cells by macrophages and NK cells [14,15]. Furthermore, mechanisms facilitating the survival and implantation of endometriotic cells may include their lower susceptibility to apoptosis [16,17] and increased adhesiveness and invasiveness [17,18,19,20]. The growth and progression of endometriotic lesions appear to be stimulated by an abrogated local estrogen release [21,22]. These phenomena are also supported by the induction of local new blood vessel formation; indeed, angiogenesis seems to play a crucial role in the etiopathogenesis of endometriosis [23].

Chromogranin A (CgA), an acidic hydrophilic glycoprotein composed of 439 amino acids with a molecular mass of 49 kDa, is a key member of the granin family [24]. CgA is mainly localized in the cell cytoplasmic electron-dense chromaffin granules, primarily in the adrenal medulla and the cells of the diffuse neuroendocrine system [25]. The human *CHGA* gene is located on chromosome 14 (14q32.12) and consists of eight exons separated by seven intronic sequences [26,27]. CgA itself may exert some biological functions [28,29]; however, it is also a precursor for a variety of biologically active peptides. The best recognized are catestatin (CST), pancreastatin (PST), vasostatins (VSs) I and II, prochromacin, chromacin, chromofungin, WE-14, and serpinin [25,28,30].

CgA and its derivatives play a role in regulating various biological activities, including angiogenesis and endothelial permeability, myocardial contractility, glucose and calcium homeostasis, and innate immunity, and exert both adrenergic and anti-adrenergic effects [30]. Their increased concentrations were reported in neuroendocrine tumors and non-malignant conditions, including organ failure and cardiovascular disease [31,32,33].

There is growing evidence that CgA and its derivatives may also be actively involved in the pathogenesis of various autoimmune and inflammatory disorders [30]. Endometriosis is associated with chronic pelvic inflammation and increased production of various autoantibodies and, therefore, may be considered an autoimmune/autoinflammatory disorder [34,35]. Accordingly, the present study aimed to evaluate serum and peritoneal fluid concentrations of CgA and its derivatives, CST and PST, and to correlate these levels with disease severity.

## 2. Materials and Methods

### 2.1. Patients

In total, 125 women, recruited from the I and II Departments of Obstetrics and Gynaecology at the Medical University of Warsaw, Poland, were included in the study. All participants gave written consent to the study, and the study protocol was approved by the Institutional Bioethical Review Board of the Medical University of Warsaw, Poland (KB/257/2016). Patients with laparoscopically and histologically confirmed endometriosis (n = 65) were included in the endometriosis group. Disease severity has been classified according to the American Society for Reproductive Medicine (rASRM) criteria [36]. The control group (n = 60) consisted of patients with no clinical symptoms or signs of endometriosis, subjected to laparoscopic examination and/or surgery for other unrelated reasons, such as dermoid/benign ovarian cyst, or infertility. All women in the endometriosis and control groups had regular menstrual cycles and did not suffer from other chronic endocrine, autoimmune, infectious, or neoplastic disorders. They were not subjected to hormonal pharmacological treatment or any other chronic medication for a minimum of 3 months before the study. The menstrual cycle phase was determined based on the date of the last menstrual bleeding.

### 2.2. Collection of Serum and Peritoneal Fluid Samples

Serum and peritoneal fluid samples were acquired during the mid-proliferative phase of the patients’ menstrual cycle (8th–10th day). Whole blood samples were routinely obtained preoperatively (immediately before operation) from fasting patients’ peripheral blood. After centrifuging at 2500× *g* (4 °C, 10 min), the obtained serum samples were stored (−80 °C) for further evaluations. During laparoscopy performed under general anesthesia, peritoneal fluid (PF) was aspirated from the cul-de-sac, before any further procedures. Specimens contaminated with blood were excluded from studies. Samples of PF were centrifuged at 2000× *g* (4 °C, 10 min), and the cell-free supernatants were stored (−80 °C) for further analyses. For both serum and PF, the procedures mentioned above were performed within one hour after collection.

### 2.3. Evaluation of CgA and Its Derivatives in Serum and PF

Concentrations of CgA, CST, and PST in serum and PF samples were measured by the specific Human Chromogranin-A ELISA Kit (E1730Hu), Human Catestatin ELISA Kit (E4996Hu) and Human Pancreastatin ELISA Kit (E0983Hu), respectively, according to the manufacturer’s instructions, using a FLUOstar Omega microplate reader (BMG Labtech, Offenburg, Germany) and initially processed with MARS Data Analysis Software 3.32 (BMG Labtech, Ortenberg, Germany). All ELISA kits used in the study originated from Bioassay Technology Laboratory, Shanghai, China.

### 2.4. Statistical Analysis

GraphPad Prism 10.2.3. software (San Diego, CA, USA) was used for the statistical analyses and graphical presentations. Parametric Student’s *t*-test or nonparametric Mann–Whitney U-test was used to determine differences between groups, where applicable. The Kruskal–Wallis test followed by Dunn’s multiple-comparison test was used to compare more than two groups. The significance of the difference between two independent proportions was calculated using the Z-ratio. The two-tailed Spearman correlation coefficient (rs) was used for performing correlation analyses. To assess the predictive power of the tested factors, a receiver operating characteristic (ROC) curve was generated, and the area under the curve (AUC) and 95% confidence intervals (95% CIs) were calculated. The differences between groups were considered significant at *p* < 0.05. The results are presented as mean ± SD or median with interquartile range. Correlations are shown as scatterplots with a regression line.

Multivariable linear regression analyses were performed using the R software (version 4.1.2, GUI 1.77, High Sierra build 8007; R Foundation for Statistical Computing, Vienna, Austria) to evaluate the association of CgA concentrations with endometriosis while adjusting for potential confounders. Serum and peritoneal fluid CgA concentrations were log-transformed (natural logarithm) to improve approximation of normality. The models included endometriosis stage (rASRM, coded as integer: 0 = control, 1 = stage I–II, 2 = stage III–IV), body mass index (BMI), and infertility status as predictors. Higher rASRM values reflect more advanced disease; the variable was entered as an integer to preserve the ordinal nature rather than treating each stage as a separate category. Regression coefficients (β), 95% confidence intervals, and *p*-values were calculated. Patients with missing data for any variable included in the model were excluded from the respective analysis. Exponentiated coefficients (exp[β]) can be interpreted as approximate fold-change in CgA per unit change of the predictor. Multiple R^2^ and adjusted R^2^ were reported to assess model fit.

## 3. Results

In patients with endometriosis, laparoscopic investigations revealed the presence of ovarian endometriotic cysts and/or superficial peritoneal lesions. No deep-infiltrating lesions were reported in this group. The demographic and clinical characteristics of participants with and without endometriosis are shown in Table 1. As seen, there were no differences in the age of the investigated groups. However, some significant differences were observed between the endometriosis and control groups concerning body mass index (BMI), parity, and infertility status.

**Figure 1:**
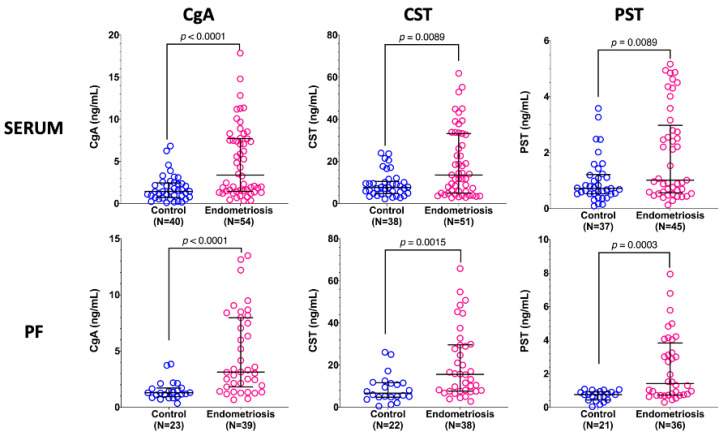
Serum and peritoneal fluid (PF) concentrations of chromogranin A (CgA), catestatin (CST), and pancreastatin (PST) in the control group and patients with endometriosis. The results are shown as medians and interquartile ranges, and *p*-values for differences between control and endometriosis groups were calculated by the Mann–Whitney U-test. N, number of samples.

The concentrations of CgA, CST, and PST were significantly higher in both the sera and PF of patients with endometriosis compared to controls (Figure 1). The concentrations of the evaluated factors in serum were strongly and significantly correlated with those seen in the PF (CgA, rs = 0.629, *p* < 0.0001; CST, rs = 0.517, *p* = 0.0004; PST, rs = 0.646, *p* < 0.0001). There was also a powerful correlation between all the tested factors in both serum (CgA vs. CST, rs = 0.811, *p* < 0.0001; CgA vs. PST, rs = 0.619, *p* = 0.0013; CST vs. PST, rs = 0.847, *p* < 0.0001) and PF (CgA vs. CST, rs = 0.902, *p* < 0.0001; CgA vs. PST, rs = 0.896, *p* < 0.0001; CST vs. PST, rs = 0.791, *p* < 0.0001). There were no significant differences in the concentrations of the tested factors between serum and PF in either the control or endometriosis groups.

**Figure 2:**
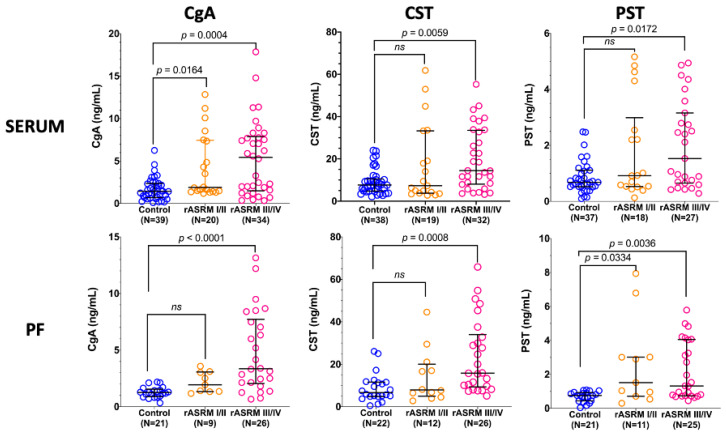
Association of the concentrations of chromogranin A (CgA), catestatin (CST), and pancreastatin (PST) in serum and peritoneal fluid (PF) with the rASRM stage of endometriosis. Results are shown as medians and interquartile ranges, and the Kruskal–Wallis test followed by Dunn’s multiple-comparison test was used to calculate *p*-values for pairwise comparisons. N, number of samples; ns, not statistically significant.

To determine whether the concentrations of the tested factors are associated with endometriosis severity, we compared their levels in serum and PF across disease stages. Significant differences were found between controls and women with advanced disease in all evaluated factors, regardless of their origin (Figure 2). In minimal/mild endometriosis, significantly higher concentrations were observed only for CgA in serum and for PST in the PF.

Spearman correlation analysis revealed a weak but significant association between the concentrations of all tested factors and the clinical stage of endometriosis, both in serum (CgA, rs = 0.391, *p* < 0.0001; CST, rs = 0.327, *p* = 0.0018; PST, rs = 0.275, *p* = 0.0119) and PF (CgA, rs = 0.509, *p* < 0.0001; CST, rs = 0.478, *p* = 0.0001; PST, rs = 0.423, *p* = 0.0011).

**Figure 3:**
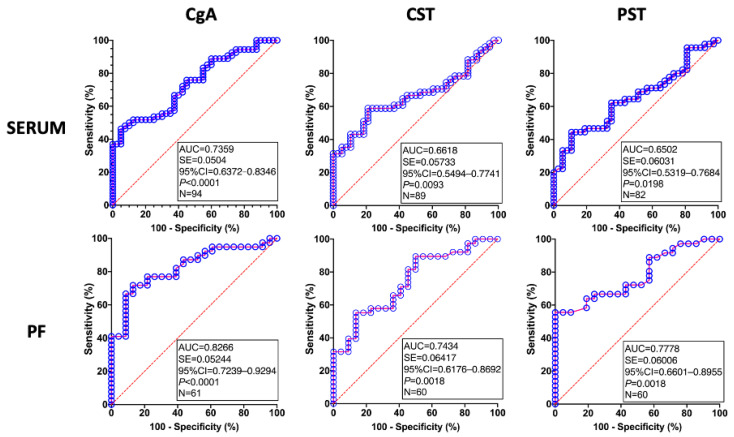
ROC analysis of chromogranin A (CgA), catestatin (CST), and pancreastatin (PST) in serum and peritoneal fluid (PF) of patients with endometriosis and control women. The area under the curve (AUC), standard error (SE), 95% confidence interval (95% CI), *p*-value, and the total number of analyzed samples (N) are given in an inset.

The results of ROC analysis of CgA and its derivatives in sera and PF from the combined population of women with and without endometriosis are presented in Figure 3. As seen, all analyses displayed high statistical significance, and the highest AUC values were observed for all factors in PF. As shown in Table 2, the Youden index and positive predictive value (PPV) and negative predictive value (NPV) for diagnosing endometriosis varied across the tested factors and their origins. The highest parameters were observed for PF CgA, with an AUC above 0.80, a Youden index of 58.75%, and PPV and NPV of 90.32% and 64.51%, respectively.

Furthermore, to evaluate the association of CgA concentrations with endometriosis while adjusting for BMI and infertility as potential confounders, multivariable linear regression analyses were performed as described in Materials and Methods section. The results are shown in Table 3. As can be seen, a significant positive association was found between PF CgA concentrations and the rASRM stage of endometriosis. This association was absent in the case of serum, where CgA was negatively associated with BMI. Removal of BMI from the regression model resulted in a significant association of serum CgA with both endometriosis and infertility.

## 4. Discussion

The results of the present study show that endometriosis is associated with increased concentrations of CgA and its derivatives, CST and PST, in serum and PF. The levels of CgA and its derivatives were predominantly elevated in endometriosis patients with moderate/severe disease, and there was a positive correlation with rASRM stage classification. This strongly argues for the role of elevated CgA and its derivatives in the pathogenesis of endometriosis.

This is a novel observation, as a relationship between endometriosis and CgA has not been reported to date. The mechanisms and clinical significance of elevated CgA and its derivatives in endometriosis remain obscure. Circulating CgA was found to be elevated in cancer [24,37], some cardiovascular disorders [28,32], and a variety of autoimmune and inflammatory disorders, including diabetes mellitus type I, rheumatoid arthritis, Crohn’s disease, giant cell arteritis, and systemic lupus erythematosus [38,39,40]. Indeed, endometriosis displays some features of an autoimmune disorder [34,35,41,42] and was reported to be associated with a variety of other autoimmune conditions [43]. It is therefore plausible that increased CgA concentrations may be related to the immune status of this disease. It is noteworthy that upregulated circulating CgA may also be associated with certain pharmacological therapies. This may be exemplified by treatment with proton-pump inhibitors (PPIs), which have been reported to increase plasma and serum CgA concentrations in patients with dyspepsia and gastroesophageal reflux [44,45]. Nevertheless, patients and control women enrolled in the present study did not have any chronic disorder. They were not receiving any chronic pharmacological therapy for a minimum of 3 months prior to the onset of the study.

The levels of circulating CgA, CST, and PST were strongly correlated. CST and PST are peptides derived from proteolytic processing of the CgA prohormone [46,47,48]. Therefore, their elevated concentrations in endometriosis are primarily due to increased CgA production. A variety of extracellular and intracellular proteases mediate proteolytic processing of CgA [46,49,50,51,52]. Interestingly, some of them, such as plasmin, were postulated to play a role in the pathogenesis of endometriosis [53].

It remains unclear whether increased concentrations of CgA and its derivatives in PF result from elevated serum levels, or vice versa. Irrespective of the origin and mechanism of increased CgA and its derivatives, they may participate in a variety of pathological processes underlying the development and persistence of endometriotic lesions. Endometriosis is related to local inflammatory reactions [54,55]. Accordingly, CgA and PST have been reported to exert a variety of pro-inflammatory effects, including the production of pro-inflammatory cytokines [30,56,57], macrophage activation [30,58], and the release of reactive oxygen species (ROS) [59], which may exacerbate the course of the disease. On the other hand, CST and other derivatives were found to exhibit immunomodulatory effects, including M2 macrophage polarization [30,60] and reduced inflammatory responses [60,61]. The latter mechanisms may facilitate the growth of endometriotic lesions and stimulate tissue repair mechanisms, leading to local fibrosis. Furthermore, CgA may modulate angiogenesis [62,63,64], a primary mechanism facilitating endometriotic lesion survival and progression [65,66]. Accordingly, it would be of interest to know whether increased concentrations of CgA and its derivatives may precede the development of the disease. However, to the best of our knowledge, there is no information on their concentrations in women at risk before puberty.

ROC analysis further confirmed that endometriosis is associated with increased circulating CgA, CST, and PST levels. Although the results of this analysis suggest that they may be considered markers of endometriosis, their use for a minimally invasive diagnosis of the disease seems to be limited and requires further investigation.

To determine whether upregulated CgA was influenced by other factors, such as BMI or infertility, we performed multivariable linear regression analysis. This analysis confirmed that increased concentrations of peritoneal CgA were associated with high-grade endometriosis. Interestingly, however, in the case of the serum CgA, this association was no longer observed, and serum CgA was found to be negatively associated with BMI. Indeed, the endometriosis group had a significantly lower BMI than the control group, consistent with previous observations that BMI may be reduced in patients with endometriosis [67,68,69,70]. The mechanism and clinical significance of this phenomenon remain unclear. CgA derivatives, CST, and PST may have an important impact on metabolism [71,72] and were also reported to be associated with lowered BMI [73]. In our opinion, this is an interesting observation that may shed new light on the association between endometriosis and energy balance and deserves further investigation.

The limitations of the present study are that body fluid concentrations of CgA and its derivatives were analyzed in the context of endometriotic lesion localization and disease severity, as judged based on rASRM classification, and did not include information regarding deep infiltrating lesions. Similarly, we were also unable to analyze the results in the context of other clinical symptoms of endometriosis, such as pain and dysmenorrhea. This information would be of interest and would support further investigations into other groups of endometriosis patients.

In conclusion, our study shows that CgA and its derivatives are upregulated in endometriosis, strongly suggesting their role in endometriosis pathogenesis. However, the origin of elevated CgA in endometriosis and the mechanisms by which CgA and its derivatives may affect the course of the disease remain unclear and require further extensive examinations.

## Figures and Tables

**Table 1:** Characteristics of patients with endometriosis and control subjects.

Characteristics		Control	Endometriosis	*p*-Value *
Number of cases (N)		60	65	
Age, years (mean ± SD)		33.19 ± 8.03	32.92 ± 5.74	0.8301
BMI, kg/m^2^ (mean ± SD)		24.61 ± 5.33	22.38 ± 3.66	0.0220
rASRM	I (minimal)/II (mild)	-	25 (38.5%)	-
	III (moderate)/IV (severe)	-	40 (61.5%)	-
Lesion localization	Ovarian	-	20 (30.8%)	-
	Peritoneal	-	25 (38.5%)	-
	Both	-	20 (30.8%)	-
Parity (mean ± SD)		1.25 ± 1.15	0.23 ± 0.44	0.0161
Infertility		20 (33.3%)	54 (83.3%)	<0.0001

* p-values for differences between mean values were calculated by Student-*t* test, whereas z-ratio was used for the calculation of the significance of the difference between two independent proportions.

**Table 2:** Diagnostic value of chromogranin A (CgA), catestatin (CST), and pancreastatin (PST) concentrations in serum and peritoneal fluid (PF) to predict endometriosis.

Investigated Factor	Sample Origin	Youden Index (%)	PPV (%)	NPV (%)
CgA	serum	41.3	92.59	56.72
	PF	58.75	90.32	64.51
CST	serum	37.77	78.95	58.82
	PF	41.62	87.5	52.77
PST	serum	33.63	83.33	56.89
	PF	55.56	100	56.76

**Table 3:** Multivariable linear regression models for log-transformed CgA concentrations in serum and peritoneal fluid (PF).

Variable	Serum log [CgA]			PF log [CgA]		
	β	*p*-Value	95% CI	β	*p*-Value	95% CI
rASRM	0.172	0.2047	−0.096–0.441	0.402	0.0135	0.088–0.715
BMI	−0.061	0.0143	−0.1102–−0.0127	−0.005	0.8874	−0.069–0.060
Infertility	0.196	0.3869	−0.2537–0.6465	0.062	0.8252	−0.508–0.633
N	71			37		
Multiple R^2^/Adjusted R^2^	0.131/0.092			0.171/0.096		

Multivariate linear regression was performed as described in the Materials and Methods section. CI, confidence interval. N, number of cases.

## Data Availability

The raw data supporting the conclusions of this article will be made available by the authors on request.

## Abbreviations

The following abbreviations are used in this manuscript:

AUC	Area under the curve
CgA	Chromogranin A
CST	Catestatin
ELISA	Enzyme-linked immunosorbent assay
NPV	Negative predictive value
PF	Peritoneal fluid
PPV	Positive predictive value
ROC	Receiver operating characteristic
rs	Correlation coefficient
SD	Standard deviation
SE	Standard error
PST	Pancreastatin
VS	Vasostatin
